# Meetings of Societies

**Published:** 1938

**Authors:** 


### Meetings of Societies

### Bristol Medico-Chirurgical Society

### SIXTY-SIXTH SESSION, 1938–1939.

THE Annual Meeting of the Society was held in the Physiological Lecture Theatre, Bristol University, on Wednesday, 12th October, 1938, at 8.30 p.m. Eighty-nine members were present.

Mr. Clifford Moore resigned the Chair to the new President, Dr. Leonard A. Moore. A vote of thanks to the retiring President was proposed by Mr. Wright, seconded by Dr. Milling, and carried with acclamation.

Dr. Leonard A. Moore then gave his inaugural address, entitled "**Medicine and the Drama.**" Professor Rendle Short proposed and Mr. Stock seconded a vote of thanks to the President for his address.

The annual reports of the Honorary Secretary, the Honorary Treasurer and the Honorary Editor of the Society's *Journal* were read and adopted.

The Society then elected the officers for the ensuing year, Dr. Charles Corfield being adopted as President-Elect. The

### BRISTOL MEDICO-CHIRURGICAL SOCIETY.

### INCOME AND EXPENDITURE ACCOUNT for the year ended 30th September, 1938.

### INCOME.

| | £ | s. | d. | £ | s. | d. |
|---|---|---|---|---|---|---|
| Members' Subscriptions .. | 282 | 19 | 6 | | | |
| Do., outstanding for 1937–1938 .. .. .. .. .. | 44 | 2 | 0 | | | |
| | | | | 327 | 1 | 6 |
| Interest on Bank Deposits .. .. | | | | 2 | 11 | 2 |
| Interest on £500 4 per cent. Consolidated Stock, less tax .. .. | | | | 14 | 15 | 0 |
| | | | | £344 | 7 | 8 |

### EXPENDITURE.

| | £ | s. | d. | £ | s. | d. |
|---|---|---|---|---|---|---|
| Grant to *The Bristol Medico-Chirurgical Journal* .. .. .. | | | | 205 | 0 | 0 |
| Librarian—Honorarium .. .. .. | | | | 15 | 0 | 0 |
| Miscellaneous Expenses— | | | | | | |
| Goodway–Refreshments | 17 | 0 | 0 | | | |
| Postages and Sundries | 11 | 0 | 0 | | | |
| Printing and Stationery | 10 | 14 | 6 | | | |
| Audit Fee .. .. .. | 2 | 2 | 0 | | | |
| Lantern and Cinematograph at Meetings .. | 4 | 4 | 0 | | | |
| University Marshal .. | 1 | 0 | 0 | | | |
| Lewis's Medical and Scientific Library— (£ s. d.) Subscription 5 10 0; Postages .. 0 17 9 | 6 | 7 | 9 | | | |
| Share of Expenses Cathedral Service .. | 2 | 14 | 0 | | | |
| Cleaning and Reframing Pictures .. .. .. | 2 | 12 | 6 | | | |
| | | | | 57 | 14 | 9 |
| | | | | 277 | 14 | 9 |
| Surplus for the year .. .. .. | | | | 66 | 12 | 11 |
| | | | | £344 | 7 | 8 |

### BALANCE SHEET as at 30th September, 1938.

### LIABILITIES AND CAPITAL.

| | £ | s. | d. | £ | s. | d. |
|---|---|---|---|---|---|---|
| Sundry Creditors .. .. | | | | 68 | 18 | 6 |
| Capital as at 30th Sept., 1937 .. .. .. | 1,067 | 13 | 10 | | | |
| *Add* Surplus for the year | 66 | 12 | 11 | | | |
| | | | | 1,134 | 6 | 9 |
| | | | | £1,203 | 5 | 3 |

### ASSETS.

| | £ | s. | d. | £ | s. | d. |
|---|---|---|---|---|---|---|
| Cash at Bank— | | | | | | |
| Current Account .. .. | 545 | 9 | 9 | | | |
| Deposit Account .. .. | 176 | 4 | 6 | | | |
| | | | | 721 | 14 | 3 |
| £500 4 per cent. Consolidated Stock at cost .. .. .. .. .. .. | | | | 437 | 9 | 0 |
| Sundry Debtors— Members' Subscriptions for 1937–1938 outstanding .. | | | | 44 | 2 | 0 |
| | | | | £1,203 | 5 | 3 |

Audited and found correct,

H. E. KEELER,

*Chartered Accountant,*

*Auditor.*

16–18 Clare Street, Bristol 1.

10*th October,* 1938.

Honorary Treasurer, Mr. A. E. Iles, and the Honorary Secretary, Mr. R. V. Cooke, were re-elected.

Professor Nixon proposed and Mr. Scarff seconded the names of the following members to serve on the Medical Library Committee: Professor Perry, Professor Drew Smythe and Professor Rendle Short.

---

The second meeting of the session was held in the Physiological Lecture Theatre, Bristol University, on Wednesday, 9th November, at 8.30 p.m.

Dr. Leonard Moore was in the Chair, and sixty-five members were present.

Mr. F. W. Willway shewed a blind patient with his Alsatian guide-dog. Dr. Bush shewed skiagrams and Dr. Fraser pathological specimens.

After discussion it was decided to hold future meetings in the Physics Lecture Theatre, Royal Fort. Professor Tyndall had kindly offered the use of his department and the service there.

Professor Hewer gave a lecture, illustrated by lantern slides, "**Syphilis in the Tropics,**" and replied to questions raised by Professor Nixon.

---

The third meeting of the session was held in the Physics Lecture Theatre, Royal Fort, on Wednesday, 14th December, at 8.30 p.m.

Dr. Leonard Moore was in the Chair, and 127 members were present.

Dr. Burgin showed skiagrams and Dr. A. L. Taylor pathological specimens.

Professor G. Hadfield gave an address on "**The Anæmias: a survey of our present knowledge,**" replying to the various questions asked by Mr. Adams, Mr. Pridie, Dr. Paul Bodman and Dr. Milling.

